# Update on Treatment of Feline Infectious Peritonitis: European Advisory Board on Cat Diseases (ABCD) Guidelines

**DOI:** 10.3390/v18040452

**Published:** 2026-04-09

**Authors:** Séverine Tasker, Andrea M. Spiri, Katrin Hartmann, Diane D. Addie, Sándor Belák, Michèle Bergmann, Herman Egberink, Tadeusz Frymus, Regina Hofmann-Lehmann, Fulvio Marsilio, Maria Grazia Pennisi, Etienne Thiry, Uwe Truyen, Corine Boucraut-Baralon, Karin Möstl, Margaret J. Hosie

**Affiliations:** 1 Bristol Veterinary School, University of Bristol, Bristol BS40 5DU, UK; s.tasker@bristol.ac.uk; 2 International Cat Care, Tisbury, Salisbury SP3 6HJ, UK; 3 Clinical Laboratory, Department of Clinical Diagnostics and Services, Vetsuisse Faculty, University of Zurich, 8057 Zurich, Switzerland; regina.hofmann-lehmann@uzh.ch; 4 LMU Small Animal Clinic, Centre for Clinical Veterinary Medicine, LMU Munich, 80539 Munich, Germany; hartmann@lmu.de (K.H.); michele.bergmann@lmu.de (M.B.); 5 Independent Researcher, 64000 Pyrénées Atlantique, France; draddie@catvirus.com; 6 Department of Animal Biosciences, Swedish University of Agricultural Sciences, SLU, Box 7023, S-750 07 Uppsala, Sweden; sandor.belak@slu.se; 7 Department of Biomolecular Health Sciences, Faculty of Veterinary Medicine, University of Utrecht, 3584 CL Utrecht, The Netherlands; h.f.egberink@uu.nl; 8 Department of Small Animal Diseases with Clinic, Institute of Veterinary Medicine, Warsaw University of Life Sciences—SGGW, 02-787 Warsaw, Poland; tadeusz_frymus@sggw.edu.pl; 9 Faculty of Veterinary Medicine, Università degli Studi di Teramo, 64100 Teramo, Italy; fmarsilio@unite.it; 10 Retired from Department of Veterinary Sciences, University of Messina, 98100 Messina, Italy; mariagrazia.pennisi@unime.it; 11 I Periodeuti ASC, 89122 Reggio Calabria, Italy; 12 Department of Infectious and Parasitic Diseases, FARAH Research Centre, Faculty of Veterinary Medicine, Liège University, B-4000 Liège, Belgium; etienne.thiry@uliege.be; 13 Institute of Animal Hygiene and Veterinary Public Health, University of Leipzig, 04103 Leipzig, Germany; truyen@vetmed.uni-leipzig.de; 14 Antech Laboratories, 31770 Colomiers, France; corine.boucraut@scanelis.com; 15 Retired from the Centre of Pathobiology, University of Veterinary Medicine, 1210 Vienna, Austria; karinmoestl@gmail.com; 16 MRC-University of Glasgow Centre for Virus Research, Glasgow G61 1QH, UK; margaret.hosie@glasgow.ac.uk

**Keywords:** FIP, feline coronavirus FCoV, remdesivir, GS-441524, molnupiravir, EIDD-2801, EIDD-1931, GC376, nirmatrelvir, prednisolone

## Abstract

Feline infectious peritonitis (FIP) is a disease arising as a result of feline coronavirus infection. It used to be regarded a fatal disease, with euthanasia commonly recommended following diagnosis due to its very poor prognosis. The availability of effective antiviral therapies, particularly nucleoside analogues such as oral GS-441524, has fundamentally changed the outlook for cats with FIP. FIP is now a treatable and frequently curable disease. In these revised guidelines, the European Advisory Board on Cat Diseases (ABCD) presents an update on the treatment of FIP, incorporating the findings of new studies including the range of available treatments (such as GS-441524, remdesivir and molnupiravir (EIDD-2801) and its active metabolite EIDD-1931), which varies globally, as well as suggestions for monitoring and prognostic indicators. Tables are used to present easy-to-find information on antiviral and supportive treatments for cats with FIP. GS-441524 is the most extensively studied antiviral for FIP with treatment success rates often exceeding 90%. Remdesivir is primarily reserved as an injectable antiviral for severely affected cats unable to tolerate oral medication; it is usually replaced by oral medication as soon as, and when, possible. Although 84-day treatment courses have historically been used, emerging evidence suggests that shorter regimens of 42 days can be equally effective.

## 1. Introduction

The European Advisory Board on Cat Diseases (ABCD) Feline Infectious Peritonitis complete guidelines were first published in 2009 [1] and comprehensively revised and published again in 2023 [2]. This review presents a further update on a section of the full guidelines describing the treatment of FIP, a field of rapidly evolving evidence on an increasing number of antiviral drugs used for FIP. The availability of predictably effective treatments for FIP has totally changed the outlook of this disease for owners, caregivers and veterinarians. There is now an alternative to euthanasia as FIP has largely become curable (see Table 1). Novel antiviral treatments act quickly, with clinical improvement within a few days, allowing for the trial (diagnostic) treatment of cats in which FIP is very likely (see the ABCD FIP Diagnostic Tool for further quick-to-access information on diagnosis [3]) without confirmation of diagnosis (e.g., cat too sick to undergo testing, testing not available, financial constraints) or while waiting for diagnostic results [4,5]. A rapid and sustained positive response to antiviral treatment is a means of supporting a diagnosis of FIP.

Antivirals are not licenced in cats and are still not available legally in all countries, complicating their use. An increasing number of countries have access to veterinary compounded GS-441524 products [6,7,8,9,10,11], and others have access to antivirals developed or available for humans, such as remdesivir or molnupiravir. In other countries, owners source antivirals themselves online, often illegally [4,12,13], but the quality and content of active ingredients in these preparations is usually unknown and varies significantly, as they are unregulated [14,15]. However, they are clearly often effective based on published studies [12]. Veterinarians are often prevented by local regulations from prescribing and administering such treatments [16,17,18], but provision of veterinary-led supportive care is usually allowed and highly recommended [19] as prompt supportive care that sometimes needs to be intensive is believed to be an important reason for the excellent responses to therapy [20,21].

Details on antiviral and supportive treatments for FIP (including administration, dosages and possible adverse effects) are given in Table 1 and Table 2, respectively, of this review as easy-to-find information. These tables should be used in conjunction with the summary information given below.

## 2. Antiviral Treatments

### 2.1. GS-441524

GS-441524 is a nucleoside analogue with published success rates ranging from 80% to 100% [20,21,22,23,24,25,26,27]; five studies have reported success rates of over 90–91% [26], 94% [25,28], 95% [21] and 100% [20] (Table 1a).

In initial studies, GS-441524, usually unregulated, was administered by subcutaneous (SC) injection, which was often painful and associated with complications [13,22,29,30]. Now oral GS-441524 preparations (usually tablets but also suspensions/liquid forms) are available and are very effective [19,20,23,25], and often cheaper and better tolerated than SC injections. Intravenous injectable preparations (usually of remdesivir, see below) are now usually reserved for very sick cats during initial treatment only that cannot tolerate oral medication [8]. A dosage of 15 mg/kg every 24 h (q 24 h) PO of GS-441524 is effective in most cats [21], not only in effusive cases but across all manifestations of FIP ([28] with over 200 additional cases reported by personal communication: A.M.S., K.H., R.H.L.). However, higher dosages of GS-441524 are used by some for cats with ocular and/or neurological signs (see Table 1a). Some cats seem to react better to twice daily dosing and dosages of 10–15 mg/kg q 12 h are safe if higher dosages are deemed necessary, especially for neurological disease and if inadequate response to lower dosage occurs.

**Figure 1:**
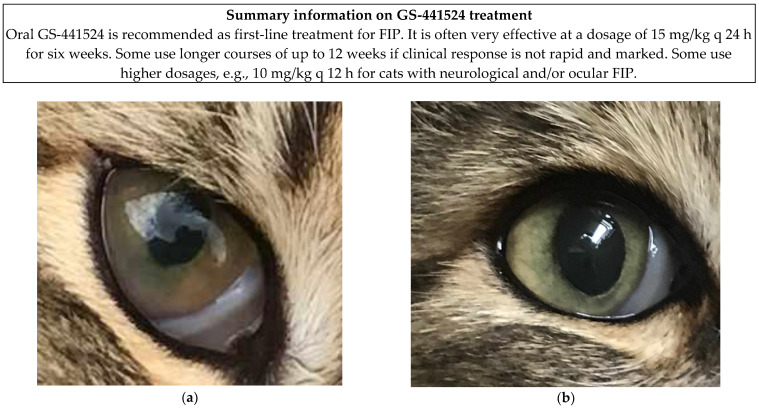
Right eye of a two-year-old male neutered Norwegian Forest cat with uveitis and keratic precipitates due to FIP prior to treatment (**a**), and following 7 weeks of oral GS-441524 and 2 weeks of topical prednisolone treatment (**b**). The full case report was published in 2020 [34]. Images courtesy of Mrs Johanna Covell-Ritchie.

Most studies in the past have used 84-day treatment courses [7,23,25,31,32,33], but shorter courses are effective (Figure 1) [19,34,35,36]. A prospective study showed that 42 days of GS-441524 PO was as effective as 84 days of treatment in cats, particularly those with effusions [21]. In another prospective bicentre study, more than 300 cats with different clinical manifestations of FIP were successfully treated with a 42-day course (this cohort included 100 cats previously described [28], with additional cases reported by personal communication: A.M.S., K.H., R.H.L.). This has important implications for access to care for cats with FIP as a shorter course reduces costs, minimises potential compliance difficulties or side effects, decreases stress associated with treatment administration and causes fewer adverse events [21].

Non-clinically significant transient adverse effects of GS-441524 include elevations in ALT [8,11] (hepatoprotectants are sometimes given but are unlikely to be needed), lymphocytosis, eosinophilia and diarrhoea [37] or vomiting [11]. An increased incidence of large cell lymphoma was observed in cats within two years after FIP diagnosis and successful GS-441524 treatment (4/202; 2%) compared with the expected incidence in the general feline population [38]. This finding might be associated with immune overstimulation due to FIP as reflected by lymphocytosis and/or lymphadenomegaly observed during and after treatment and/or a long-term complication after GS-441524 treatment. Very rarely, GS-441524 uroliths have been reported, but these appear to have occurred largely when unregulated injectable products have been used. Such products might have contained higher amounts of GS-441524 than are normally used for treatment [39,40].

### 2.2. Remdesivir

Remdesivir is a nucleoside analogue and the prodrug of GS-441524 [41]. It is usually expensive. A human-licenced preparation is available, as well as veterinary compounded products, in some countries. Its usage for the treatment of FIP in cats is allowed by the European Union [42]. Remdesivir has usually been injected, either intravenously or SC, but SC administration is often painful (but can depend on the remdesivir preparation) and so, when remdesivir has to be used, for example, at the start of treatment, it is usually given intravenously [43] (Table 1a). Oral GS-441524 treatment for FIP is now favoured to avoid painful SC injections. However, injectable (intravenous) treatment might be necessary when remdesivir is the only antiviral available and/or the cat is unable to tolerate oral medication (e.g., due to being very sick at the beginning of treatment with severe neurological disease or suspected reduced gastrointestinal motility and absorption) [6,8,9,11,36]. Remdesivir can also be used orally in countries where a legal compounded oral formulation is available [10,31]. Intravenous injectable preparations (usually of remdesivir, see below) are now usually reserved for very sick cats.

### 2.3. Molnupiravir

Molnupiravir (EIDD-2801) is another oral nucleoside analogue prodrug, which is rapidly hydrolysed to EIDD-1931 [44], its active metabolite [45] (Table 1a). It acts differently to remdesivir in that its action (and that of EIDD-1931) induces lethal mutagenesis in the virus [46]. It is licenced in the USA as a human drug, but not in the EU; it can need compounding to enable accurate dosing for cats. It has shown good results as a first-line treatment, effective in 72% [47], 77% [48], 86% [27], and 91% [49] of cats, and as a rescue agent, effective in 86% to 100% [47] of cats after relapse to previous treatment (often GS-441524). Following relapse of 7/56 cats at 9–99 days after discontinuing initially effective first-line molnupiravir, a second course of molnupiravir at a higher dosage successfully induced remission [48].

Rules vary in different countries as to whether molnupiravir can be used in cats. It is usually more affordable than GS-441524 or remdesivir. The duration of treatment needed has not been defined but studies have usually described 84-day treatment periods [27,33,44,47,48,49,50]. Care is required for handling of molnupiravir [48,51] due to its mutagenic properties from human studies [46,52].

Response to molnupiravir might also be slower than to GS-441524 [47]. Side and adverse effects can be severe (Table 1a), including severe leukopenia at very high dosages [45] and neutropenia [47], but also non-clinically relevant increase in ALT [27,47,48,50], nausea, hypersalivation [48] and vomiting or diarrhoea [49]. The slower response and its potentially severe adverse effects, including mutagenic properties, suggest that other nucleoside analogues, such as GS-441524, should be used in preference, if available and possible.

### 2.4. EIDD-1931

EIDD-1931 (β-d-N4-hydroxycytidine-NHC), a metabolite of molnupiravir, is also an oral nucleoside analogue that is available in some countries (Table 1a). As for molnupiravir, there are concerns regarding its mutagenicity [46], suggesting that other nucleoside analogues, such as GS-441524, should be used in preference, if available. One study [53] described the use of EIDD-1931 in nine cats (six with effusions), for 84 days in eight cats and 98 days in one cat. All cats responded to treatment although one cat relapsed twice after treatment completion, responding to higher dosages of EIDD-1931 before developing lymphoma, resulting in euthanasia. Response to EIDD-1931 treatment appears to be slower, and adverse effects more common, than to GS-441524 or remdesivir. Side and adverse effects include hyporexia, transient neutropenia, elevated ALT and broken whiskers [53]. EIDD-1931 should probably be reserved for cats that do not respond to GS-441524, although it is more affordable and available in some countries.

### 2.5. GC376

GC376 is an injectable protease inhibitor antiviral that has been used alone successfully for the treatment of FIP in one study [54], and in combination with GS-441524 in another study [55] (Table 1b). Dentition adverse effects were reported [54]. No legal preparations are currently available although it is hoped that a cat-licenced product will be available in the future, making this treatment accessible.

### 2.6. Nirmatrelvir

Some veterinarians have used the protease inhibitor antiviral oral nirmatrelvir, given with ritonavir (personal communication: Sally Coggins and Richard Malik) (see Table 1b) in refractory cases, as adjunct treatment to a nucleoside analogue [53]. Nirmatrelvir treatment, alongside GS-441524 in six cats and molnupiravir in one cat, and given alone to one cat, was reported in a retrospective study that was focused on treatment outcomes [56]. Dosages varied and all seven cats given it as adjunct treatment survived, whilst the one cat given nirmatrelvir alone died within two weeks of starting treatment. It is usually expensive. More data are required.

### 2.7. Recombinant Feline Interferon-Omega (rfIFN-ω)

rfIFN-ω is licenced for use in cats (not for the treatment of FIP but for the general supportive treatment of retrovirus-infected cats) in some countries (Table 1b). It is not effective on its own for the treatment of FIP [57], but it has been used following antiviral therapy with GS-441524 to prevent relapse by some [19,34]. However, controlled studies are needed to confirm efficacy of, and need for, rfIFN-ω, as many studies have shown excellent survival following nucleoside analogue (including GS-441524) treatment without follow-up rfIFN-ω [6,8,9,20,22,23,25,29].

### 2.8. Mefloquine

Oral mefloquine is an affordable human-licenced anti-malarial treatment that has been tested on healthy cats [58] but no controlled FIP treatment studies exist, although its use is mentioned as adjunct treatment in some studies [47,53,59] (Table 1b). It might maximise effectiveness of antivirals if relapse is suspected, or in cases where other more effective antivirals cannot be used due to cost or availability, but more data are required. It is given with food to avoid vomiting as a side effect.

### 2.9. Other Antivirals

Other antiviral agents, such as mesenchymal stem/stromal cells (MSCs), which are currently being investigated and show promise [60,61], exist but are not described in this treatment update.

## 3. Immunomodulatory Treatments

Immunomodulatory therapies are no longer considered as sole treatments for FIP as they are ineffective in comparison to the antiviral treatments described earlier. Glucocorticoids are sometimes indicated combined with antivirals and the use of polyprenyl immunostimulant (PI) has been described in a few studies.

### 3.1. Glucocorticoids

Glucocorticoids can act as anti-inflammatories or immunosuppressants depending on dosage used. The median survival time of FIP cats treated with prednisolone was only eight days in one study [57] and 31 days in cats with suspected effusive FIP [62]. Although it is suggested that glucocorticoid treatment is associated with a poorer FIP outcome when used concurrently with other treatments (e.g., such as rfIFN-ω [19]), glucocorticoid use is helpful alongside antivirals in some situations, e.g., topical steroids for uveitis [34,63], anti-inflammatory prednisolone (1 mg/kg/day) for severe FIP-associated neurological signs [64,65,66] or immunosuppressive prednisolone (1–2 mg/kg/day) for FIP-associated IMHA [66,67], in cases where concurrent haemotropic mycoplasma infection has ideally been ruled out. In one study prednisolone was used in cats with neurological or ocular signs, at a tapering dosage starting at 1 mg/kg q 24 h, terminating before the end of the molnupiravir treatment [48].

### 3.2. Polyprenyl Immunostimulant

PI is an immunostimulant that might be helpful in the treatment of a few cats with FIP without effusions, especially in cats with haematocrit and/or albumin to globulin (A:G) ratios that are normal or that increase with treatment, although response to treatment is slow, over several months (normalisation times are ~182 days for haematocrit and ~375 days for the A:G ratio) [68]. It has been found that concurrent systemic glucocorticoid treatment should be avoided with PI, as this worsens prognosis [63] but topical glucocorticoid treatment can be used with PI in ocular FIP uveitis [63]. Reported dosages are 3 mg/kg PO three times a week or q 48 h [19,63,68,69]. Some cats are changed to a maintenance dosage of 3 mg/kg PO once or twice a week after one year of treatment [65].

### 3.3. Other Immunomodulators

Anti-TNF-α antibody blocks TNF-α that is involved in exacerbating clinical signs of FIP [70,71,72]. Some efficacy of anti-TNF-α antibodies has been reported in small studies of experimental-induced FIP; in one placebo-controlled study [73], and in another uncontrolled study alongside itraconazole treatment [74] in which two of three cats with FIP improved.

**Table 1:** Antiviral drugs for FIP treatment. ALT indicates alanine aminotransferase, IV indicates intravenously, PO indicates orally, rfIFN-ω indicates recombinant feline interferon-omega, SC indicates subcutaneously. (**a**) Nucleoside analogue antivirals; (**b**) Non-nucleoside analogue antivirals. Note: Text in bold is to highlight charactertistics or usage of the described antivirals, to allow for easier visual use of the table. For all antiviral treatments, it is important to ensure the dose given to the cat preserves appropriate dosage if/when weight gain occurs because of recovery, e.g., in growing kittens and adults that have had weight loss. If the dose is not adjusted, underdosage occurs, which can be associated with disease relapse [9]. Accurate weight recording is also important to monitor response to treatment [22]. It is also important to note that unregulated antivirals are of unknown composition and concentration of active ingredient, making interpretation of ‘given’ doses difficult [14,15]. Alongside antiviral treatment, individualised analgesia and supportive care are essential for successful treatment of FIP; for further details on supportive treatment see text and Table 2.

(a) Nucleoside Analogue Antivirals				
Drug	GS-441524	Remdesivir (GS-5734)	Molnupiravir (EIDD-2801)	EIDD-1931
**Derivative**	Metabolite of remdesivir (GS-5734).	Prodrug of GS-441524.	Prodrug of EIDD-1931.	Biologically active metabolite of molnupiravir.
**Mechanism of action** [75]	Functions as an alternative substrate for viral RNA synthesis resulting in RNA chain termination.	Its metabolite functions as an alternative substrate for viral RNA synthesis resulting in RNA chain termination.	Its metabolite functions as an alternative substrate for viral RNA synthesis resulting in lethal viral mutagenesis.	Functions as an alternative substrate for viral RNA synthesis resulting in lethal viral mutagenesis.
**Regulatory status, galenic forms, handling**	Regulated compounded veterinary preparations available in some countries. Where regulated sources are not legally available, owners can procure unregulated preparations themselves, of variable quality and content [14,15], although treatment with such preparations is still often successful [12]. Oral (veterinary compounded) products, e.g., flavoured quartered 50 mg tablet and/or flavoured 50 mg/mL suspensions/liquids/pastes and/or microcapsules available. Unregulated preparations are typically injectable or oral tablets/capsules of varied formulations.	Available as licenced preparation for human use (Veklury^®^, Gilead Sciences Inc., Foster City, CA, USA) or regulated compounded veterinary preparation. Injectable (veterinary compounded) remdesivir is often made up at 10 mg/mL whereas Veklury^®^ remdesivir is a 100 mg/vial, which is reconstituted with sterile water for injection to 5 mg/mL remdesivir. Oral (veterinary compounded) available in 60 mg, 90 mg and 120 mg capsules in some countries [10].	Available as licenced preparation for human use (Lagevrio^®^, Merck & Co., Inc. [Rahway, NJ, USA and Kirkland, QC, Canada] or Merck Sharp & Dohme, MSD [Haarlem, The Netherlands]) in some countries, that can be prescribed to cats in some countries. Designated as an antimicrobial reserved for human use only in European Union (EU) in 2022 (although not licenced for human usage) [76]. Concerns exist regarding virus mutagenesis potentially leading to more virulent or drug-resistant viral variants. Concerns also exist regarding host mutagenesis. Oral Lagevrio^®^ available as 200 mg capsule that needs division into appropriate doses but care needed regarding handling due to mutagenic properties [51] from human studies [46,52]. Wear gloves.	Regulated compounded veterinary preparation available in some countries. Oral (veterinary compounded tablets), e.g., flavoured quartered 60 mg tablet, or capsules. Like molnupiravir, concerns exist regarding virus mutagenesis potentially leading to more virulent or drug-resistant viral variants. Concerns also exist regarding host mutagenesis [52]. Wear gloves.
**Routes of administration**	PO primarily as effective and avoids painful injections. SC injection less commonly used as can be painful. If oral medication cannot be tolerated, injectable remdesivir is used initially as this can be given IV (see next column).	IV injection primarily, as SC injection can be painful. PO available in some countries.	PO.	PO.
**Dosage**	**Oral:** 15 mg/kg q 24 h often used [21]. Some adopt higher dosages for cats with ocular or neurological signs (20 mg/kg q 24 h or 10 mg/kg q 12 h) [2,8,36]. Some have used up to 15 mg/kg q 12 h if inadequate response to lower dosage.	**Injectable:** IV usually given rather than SC, due to pain on SC injections. Varied dosages reported: 6–20 mg/kg q 24 h [6,9] or 15–30 mg/kg q 24 h [10] or 15 mg/kg q 24 h unless ocular or neurological signs are present, in which case 20 mg/kg q 24 h used. IV remdesivir usually given slowly; over 30 min–2 h as a constant rate infusion, can be diluted in saline. PO GS-441524 usually favoured unless injectable remdesivir is the only antiviral available and/or is needed at the start of treatment (until switch to PO GS-441524) if the cat cannot tolerate oral medication. **Oral:** 25–30 mg/kg q 24 h given for effusive FIP [10,31] (although many cats in one study [10] received injectable remdesivir IV or SC 15–30 mg/kg q 24 h at the start treatment). The poorer survival of cats with non-effusive FIP [10] prompted the suggestion of 30 mg/kg q 24 h.	**Oral:** 10–20 mg/kg q 12 h [27,48,50] or 10–15 mg/kg q 12 h [49,51], or 15 mg/kg q 12 h [47]; lower dosages usually used for FIP with effusions without ocular or neurological signs whilst higher dosages used when ocular and/or neurological signs present.	**Oral:** 15 mg/kg q 12 h [53] for most cases although 20 mg/kg q 12 h recommended for neurological cases; up to 25 mg/kg q 12 h used to treat suspected relapses in a cat previously given a lower dosage of EIDD-1931 [53].
**Administration considerations**	PO: Ideally given on an empty stomach, at least 30 min before food, but mixing GS-441524 (e.g., crushing tablets) in a small amount of a treat is possible and giving a small amount of food with the GS-441524 can prevent vomiting [2]. SC injection less commonly used as can be painful.	Compliance often problematic with painful SC injections with some formulations; IV preferable to reduce pain. PO usually given without food [10].	Has been given mixed with food [47] or with small amounts of food or treats [48]. Always given q 12 h.	Few data available but has been given on empty stomach.
**Duration of treatment**	Most studies have used 84 days [7,11,23,25,31,32,33], with duration and/or dosage increased (by 5 mg/kg/day) if clinical signs and serum biochemistry do not normalise [8,9,11], although some have used shorter courses [19,34,35,36]. A prospective randomised controlled study of cats treated with 15 mg/kg q 24 h PO (most had effusions), found that 42 days was as effective as 84 days, with a rapid response to treatment and normal clinical pathology by 42 days, and 95% survival [21]. In another prospective bicentre study, more than 300 cats with different clinical manifestations including neurological forms of FIP were successfully treated with a 42-day course (this cohort included 100 cats previously described [28], additional cases reported by personal communication: A.M.S., K.H., R.H.L.). Before stopping treatment, clinical signs, clinical pathology changes and elevated AGP concentrations should have normalised; normal AGP two weeks apart before stopping treatment is encouraging [19,77]; see Section 6. Treatment Monitoring in text.	Studies have used 84 days with injectable, PO or mixed treatments [6,9,10,31]. Monitoring of treatment duration not described but it can be that, as for GS-441524 treatment, normal AGP two weeks apart before stopping treatment is encouraging [19,77].	Studies have usually used 84 days [27,33,44,47,48,49,50,51]. Longer 126-day treatments have been used if abnormalities remained at 84 days [48]. Monitoring of treatment duration not described but it can be that, as for GS-441524 treatment, normal AGP two weeks apart before stopping treatment is encouraging [19,77].	Limited data but 84 days usually used (occasionally longer, e.g., 98-days) [53]; response to treatment seems to be slower than for GS-441524 or remdesivir suggesting 84-day treatment courses can be justified. Monitoring of treatment duration not described but it can be that, as for GS-441524 treatment, normal AGP two weeks apart before stopping treatment is encouraging [19,77].
**Survival/** **success rates**	80% in cats with varied forms of FIP [27], 81%, mainly in cats with effusions [22], 82% in cats with effusions [23], 83% in cats with varied clinical manifestations of FIP [24], 85% in cats with ‘mixed’ effusive/non-effusive FIP [25], 91% in cats with, and without, effusions [26], 94% in cats with FIP without effusions [25,28], 95% [21] and 100% in a prospective study of 18 cats [20]. Improvement in 88% (many cats still on treatment at time of writing) [12] reported using owner-reported data. Some treatment studies excluded moribund or very severely affected cats, which might have influenced reported survival rates [20,33]. Reversal of FIP-associated myocarditis with PO GS-441524 and symptomatic treatment with cardiovascular drugs also reported [78].	Protocols using initial remdesivir (IV preferably) then preferably PO GS-441524 (but SC remdesivir also) gave 86% survival at six months in 28 cats with effusive or non-effusive FIP [9]. Remdesivir (IV and/or SC) subsequently treated with GS-441524 PO (24 cats), or without GS-441524 (six cats), was associated with survival of 96%, and 33%, respectively, of 30 cats with effusive or non-effusive FIP [6]. Compounded PO remdesivir was as effective as PO GS-441524, with 77% survival in effusive FIP [31]. Remission was achieved in 75% of cats given a compounded PO remdesivir formulation, although 16 of these 22 cats received injectable remdesivir at the start of treatment [10]. Cats with non-effusive FIP were less likely to survive than cats with effusions [10].	**As first-line treatment**: Survival rates of 72% [47], 77% [48], 78% [50], 80% [51], 86% [27], 91% [49] and 100% (but 25% needed a second molnupiravir treatment) [45]. One study [33] reported less efficacy than GS-441524 in non-effusive FIP. **As rescue treatment**: 86% for molnupiravir after GS-441524 or remdesivir (median of seven days) [47], and 92% [45] and 100% after relapse following GS-441524 [45] or GS-441524 and/or remdesivir and/or mefloquine treatment [47]. A 100% response was also seen using a higher dosage in cats that had relapsed 9–99 days following stopping effective first-line molnupiravir [48]. Comparative studies between molnupiravir and GS-441524 [27,51], and between molnupiravir and GS-441524 or remdesivir [47], have shown no significant differences in treatment outcome.	In nine cats with FIP (six with effusions, three without), 100% survival, although one cat that relapsed responded to repeat EIDD-1931 at higher dosages [53] but the cat was euthanised due to lymphoma. Survival rate at a year was therefore 89% (8/9 cats).
**Possible side and adverse effects**	Non-clinically significant transient elevations in ALT [8] (although some suggest this can be due to the FIP [9,37]), lymphocytosis [26,37] and eosinophilia [37] common. Diarrhoea or vomiting and possible kidney disease [11,21,37]. Very mild Heinz body-associated haemolytic anaemia reported in two (of 40) cats during GS-441524 treatment [37]. An increased incidence of large cell lymphoma was observed in cats within two years after FIP diagnosis and successful GS-441524 treatment (4/202; 2%) compared with the expected incidence in the general feline population [38]. This finding might be associated with immune overstimulation due to FIP as reflected by lymphocytosis and/or lymphadenomegaly observed during and after treatment and/or a long-term complication after GS-441524 treatment. Rare reports of GS-441524 urolithiasis (usually with unregulated injectable preparations) [39,40]. Folded ears reported in two cats treated with unregulated preparations [27]. SC injections often painful [15], creating ulcerated skin lesions [22].	Non-clinically significant transient elevations in ALT [6] common. SC injection pain (especially with more acidic preparations), although possibly less painful than SC GS-441524 [15].	Folded ears [45,48], broken whiskers and severe leukopenia at high dosages [45], neutropenia [47,48], non-clinically significant increase in ALT [27,47,48,50], nausea, hypersalivation [48] often necessitating maropitant treatment, and vomiting or diarrhoea [49].	Non dose-dependent transient neutropenia (three cats, can be severe, e.g., 0.1 × 10^9^/L in one cat] but resolves after three, four or six weeks of EIDD-1931, with no adjustment of dosage or stopping of treatment needed), non-clinically significant increase in ALT (four cats, persistent in one of these), broken whiskers (one cat) and suspected treatment-induced hyporexia (six cats) [53]. Adverse effects appear to be more common than with GS-441524 or remdesivir [53].
**Additional notes**	Varied pricing. Not known if PO daily doses are best given q 24 h or divided q 12 h (as is more often done for the higher dosages, e.g., 20 mg/kg/day) [36]. Absorption varies from cat to cat, and it might be that administration q 12 h (or even q 8 h) is more effective in some cats (personal communication: Danièlle Gunn-Moore, based on therapeutic drug monitoring [79]). However, many studies document success with 15 mg/kg q 24 h PO [21].	Expensive, especially licenced preparation for human use.	Affordable. Many cats successfully treated as outpatients [50]. Response to molnupiravir is slower than to GS-441524 (or remdesivir) [47], and this, together with its adverse effects including mutagenic properties, mean that the recommendation is to use GS-441524 in preference to molnupiravir, if available.	Affordable. Until more data available, best reserved for cases that do not respond to, or relapse with, GS-441524, or when EIDD-1931 is the only antiviral available.
**(b) Non-nucleoside analogue antivirals**				
**Drug**	**GC376**	**Nirmatrelvir**	**rfIFN-ω**	**Mefloquine**
**Mechanism of action**	Inhibits 3C-like protease [80,81].	Inhibits 3C-like protease [82]. Promising results in vitro [82,83,84].	Immune-modulating antiviral which inhibits FCoV replication in vitro [85].	Mechanism of antiviral effect not known but inhibits FCoV replication in vitro as small molecule inhibitor [86] and as nucleoside analogue [87].
**Regulatory status, galenic forms, handling**	Not commercially available, but licencing for FIP treatment is being pursued. Owners can purchase unregulated preparations themselves of variable quality and content; in one study [14], none of the five sources of GC376 treatment contained any GC376.	Available as licenced preparation for human use (Paxlovid^®^, Pfizer [New York, NY, USA]) in some countries. Nirmatrelvir has very short half-life, so is given with ritonavir to slow down nirmatrelvir metabolism by inhibiting the cytochrome P450 pathway; Paxlovid^®^ contains both nirmatrelvir (150 mg) and ritonavir (100 mg) tablets to be given together.	Licenced for cats in some countries for the general supportive treatment of feline retroviral disease. Available as lyophilisate in, e.g., vials containing 5 million or 10 million units, with 1 mL solvent for suspension for injection.	Available as licenced preparation for human use (e.g., Lariam^®^, Roche [Basel, Switzerland]). Oral Lariam^®^ available as 250 mg tablet. Generic formulations also available.
**Routes of administration**	SC.	PO.	SC (licenced route). PO.	PO.
**Dosage and duration of treatment**	In 20 cats without neurological signs, 15 mg/kg SC q 12 h for 84 days [54]. Combined GS-441524 and GC376 treatment, at dosages of either 2.5 or 5 mg/kg SC q 24 h of GS-441524 and either 10 or 20 mg/kg SC q 12 h of GC376 was given for 28 days [55]. Further field studies required.	Said to be useful in refractory (especially neurological) cases when added to nucleoside analogue treatment such as GS-441524, molnupiravir or EIDD-1931 (personal communication: Sally Coggins) [53,56]. Seven cats given nirmatrelvir as adjunct treatment survived, whilst the one cat given nirmatrelvir alone died within two weeks of starting treatment. Adjunct nirmatrelvir treatment dosages ranged from 7.4 to 18.8 mg/kg with durations of around four to seven weeks [56]. For a 4–5 kg cat: 75 mg nirmatrelvir and 25 mg ritonavir q 12 h PO for ~20 days (personal communication: Richard Malik). More data are required.	Two off-licence dosing regimes have been used in FIP: 10^6^ units/kg SC or PO q 48 h for up to five doses, and then twice a week until rfIFN-ω treatment is stopped [19]. 10^5^ units per cat SC or PO q 24 h for the duration of rfIFN-ω treatment (which has varied from days to months) [19,34]; 0.1 mL of previously diluted stock solution containing 10^6^ units of rfIFN-ω is diluted again with 4.9 mL of saline diluent so that 0.5 mL of the 5 mL new stock solution yields 10^5^ units.	62.5 mg/cat PO 2–3 times (depending on size of cat) a week (personal communication: Richard Malik) or 10–12.5 mg/kg twice a week [59], 20–25 mg/cat PO q 24 h or 4 mg/kg PO q 24 h [47].
**Administration considerations**	SC injections can cause transient stinging [54]. Has been used in combination with other antivirals.	Nirmatrelvir and ritonavir given together in a gelatine capsule before a meal (personal communication: Richard Malik). Has been used with nucleoside analogue treatments (personal communication: Sally Coggins).	Only SC route is licenced. Used by some to maintain remission after other antiviral treatment [19,34] but controlled studies required to confirm the addition of rfIFN-ω is needed as many studies show excellent survival using nucleoside analogue (including GS-441524) treatment alone [6,8,9,20,22,23,25,29].	Given with food to reduce the risk of nausea and/or vomiting as they are common. Has been used where other more effective antivirals cannot be used due to cost or availability, although efficacy alone likely limited (personal communication: Richard Malik, Sally Coggins, Jacqueline Norris), or as adjunct treatment [47,59].
**Survival/ success rates**	Six of 20 cats [54] and 1/1 cat [19]. Combined GS-441524 and GC376 treatment was effective in 98% of 46 FIP cats with and without effusions [55].	No data yet available but has been used for refractory cases with success (personal communication: Sally Coggins).	Not effective in one placebo-controlled study [57] but cats also given high-dose glucocorticoids; might be more effective in the absence of glucocorticoids [19]. In an uncontrolled study [88], eight of 12 treated cats survived at least two years or remission, but FIP was not confirmed.	Has been used as adjunct treatment for FIP and/or to maintain remission after other antiviral treatment (personal communication: Richard Malik, Sally Coggins, Jacqueline Norris) but no studies yet other than its use with other treatments in one retrospective study evaluating molnupiravir treatment [47] and another looking at shorter courses of treatment after high-dose induction with IV remdesivir and/or PO GS-441524 [59].
**Possible side and adverse effects**	Transient stinging on injection and retarded development or the abnormal eruption of permanent teeth [54].	No data yet available	Causes vomiting if not given with food but generally appears safe in healthy cats.	Nausea and/or vomiting is common so given with food but may still require maropitant and/or ondansetron.
**Additional notes**	Resistance to GC376 reported [83] in one experimental in vivo study. More data required.	Ritonavir might interfere with the metabolism of drugs processed by cytochrome P450, so check for potential interactions. Expensive.	Affordable but likely requires additional antiviral treatments. Further studies needed.	Affordable but likely requires additional antiviral treatments. Field studies required both alone and in combination with other antivirals to evaluate if useful to maintain remission.

## 4. Supportive and Concurrent Disease Treatment

Veterinary supportive care, which should be prompt and intensive, is particularly important in the recovery of cats that are very sick due to FIP [20,21]. Supportive care treatments are outlined in Table 2. However, veterinary support might not be sought by owners who have obtained antiviral drugs illegally for their cats as veterinarians are unable to advise, obtain or prescribe illegal drugs. It is possible and important for vets to give supportive care to cats in this situation for welfare reasons. Documentation in the medical records to confirm there has been no veterinary involvement in the illegal drug procurement or administration is recommended.

Stress should be minimised throughout care, adopting feline-friendly techniques [89,90]. Analgesia is important to consider as cats with FIP can be in pain due to their disease, e.g., pleuritis, peritonitis, uveitis, glaucoma, ileus, raised intracranial pressure, meningeal inflammation. Additionally, diagnostic procedures can also elicit pain, e.g., needle or biopsy samples, centesis procedures. Pain scoring should be carried out and appropriate analgesia such as opioids, non-steroidal anti-inflammatory drugs (NSAIDs, if no contraindications) (Table 2) provided [91]. Other supportive care should be provided as needed, e.g., intravenous fluids, appetite stimulants, antiemetics, and blood transfusions [8,92].

If a pleural effusion is causing dyspnoea, therapeutic drainage is indicated to provide relief to the cat so that breathing improves; the volume removed will depend upon the volume present and how dyspnoeic the cat is. Therapeutic drainage of ascitic fluid is only indicated if the degree of abdominal effusion is large enough to be compromising respiration, and then only enough to relieve this should be drained.

Antivirals will help control intraocular uveal inflammation in uveitis [34,63,93] (Figure 1) but other specific topical treatments might be needed, such as prednisolone acetate, NSAIDs and cycloplegics for miosis. Systemic prednisolone treatment has been suggested for its anti-inflammatory effect for severe posterior uveitis or panuveitis [94] but is more commonly given for neurological or immune-mediated disease signs than uveitis [93]. Ocular improvements start within 7 days of treatment and can take several weeks to resolve [93,94]. Of note is that posterior uveitis changes might only become apparent once an anterior uveitis has resolved enough to allow fundic examination [93]. If uveitis is present, it is important to regularly monitor intraocular pressure too.

If seizures occur in neurological cases of FIP, anti-seizure medication is required [8], such as levetiracetam and/or phenobarbitone [95]. Short-term prednisolone can be beneficial for cats with severe FIP-associated neurological signs in the early days of antiviral treatment [64,66], including hydrocephalus [96].

In cats that are hypoglycaemic, dextrose supplementation can be indicated [6,97] and hypotension might need to be treated with vasopressors. A viral sepsis could be occurring in very sick cats with severe hypovolaemia, dehydration, hypoglycaemia, persistent hypotension despite fluid resuscitation and when pressor support is not completely effective. More experience with the treatment of such cases is required but they seem to carry a poor prognosis [6,21].

IMHA is now recognised to occur in association with FIP [67] and most of these cats need glucocorticoids despite their FIP responding well to antiviral treatment [11,66,67]. A low prednisolone dosage of 1 mg/kg q 24 h PO can be used, tapering once there has been a response to treatment. Ideally, haemotropic mycoplasma infections should be ruled out too. Blood transfusions [98] might be required [8,21,36,67,99,100] for severe anaemia.

Myocarditis associated with FIP, and associated clinical signs, have now been described in several case reports and studies; these cats can respond to combined antivirals such as GS-441524 and appropriate medical treatment for CHF (e.g., furosemide, pimobendan), arrhythmias, and prophylactic antithrombotic treatment [78,101,102]. Completely reversed echocardiographic changes have been reported following effective treatment of the CHF and FIP [78,102].

Concurrent FeLV and/or FIV infection has not affected the response to GS-441524 treatment [5,28,103], nor have feline calicivirus or feline herpesvirus infections [28], chronic inflammatory disease [5] or renal lymphoma [104], although management of those coinfections or diseases is likely to be needed. Of note is that three cats with confirmed FIP from a prospective bicentre study did not respond successfully to GS-441524 treatment; toxoplasma-associated disease was diagnosed at postmortem examination and was considered the likely cause of failure to recover in these cats (personal communication: A.M.S., K.H., R.H.L.).

**Table 2:** Supportive treatments that have been used in cats with FIP. Text in bold is to highlight charactertistics or usage of the described antivirals, to allow for easier visual use of the table. NSAID indicates non-steroidal anti-inflammatory drug, SC indicates subcutaneously, PO indicates orally, CRI indicates constant rate infusion. Alongside antiviral treatment, individualised analgesia and supportive care are essential for successful treatment of FIP.

Drug	Comments	ABCD Recommendation in FIP
**Opioid** **analgesics**	Pain in FIP due to pleuritis, peritonitis, uveitis, glaucoma, ileus, raised intracranial pressure, meningeal inflammation, etc. Pain scoring important.	Examples include methadone, buprenorphine. For dosages see appropriate text [91].
**NSAID** **analgesics**	See above.	Meloxicam has been used in FIP [19,105]. Do not use NSAIDs in dehydration/hypotension; care in renal disease or anorexic cats. Associated with worsening acute kidney injury in one cat with FIP [6]. For dosages see appropriate text [91].
**Metamizole**	Metamizole (dipyrone) is a non-opioid analgesic, antipyretic and spasmolytic drug licenced for use in cats in some countries.	Used in place of NSAIDs in some countries. Has been used in FIP [20]. Adverse effects include hypersalivation and vomiting. For dosages see appropriate text [91].
**Gabapentin**	Anxiolytic/analgesic/sedative which can help if SC injections that cause pain (e.g., remdesivir) are needed, when oral GS-441524 cannot be given, and it is not possible to give by another route such as IV. Can be used to reduce stress associated with vet visits (e.g., if needed for treatment monitoring) alongside other cat-friendly principles [106] and approaches [90]. Not licenced.	Has been used successfully with FIP [7,8,9,13]. Adverse effects can include sedation and ataxia. Doses that have been used in healthy cats were 50 or 100 mg (can be up to 200 mg if required but start at low dose initially) per cat PO [90]. In cats sick with FIP, lower dosages might be adequate, e.g., 10 mg/kg around two hours before SC injection, with lower dosages recommended if renal disease is present [107].
**Pregabalin**	Anxiolytic/analgesic/sedative which can help if SC injections that cause pain (e.g., remdesivir) are needed (when oral GS-441524 cannot be given, and it is not possible to give by another route such as IV). Can be used alongside cat-friendly principles and approaches.	Available as oral solution of 50 mg/mL. Dosage of 5 mg/kg PO; administer single dose around 1.5 h before SC injection; may be administered directly in mouth or mixed with small amount of food.
**Mirtazapine**	Appetite stimulant/anti-nausea medication which is used for prevention and treatment of vomiting and nausea and as appetite stimulant; can be given as a trial if nausea suspected.	Has been used in anorexic cats before/ during treatment [6,8,19,31]. Note that efficacious antiviral treatment (e.g., GS-441524) usually causes a rapid return of appetite. Given at 2 mg/cat PO or as transdermal q 24 h (q 48 h if renal/hepatic involvement of FIP) [92].
**Maropitant**	For prevention and treatment of vomiting and nausea. SC injection can be painful.	Has been used as a supportive treatment [6,8,20,48]. Dosage is 1 mg/kg SC, IV or PO q 24 h [92].
**Metoclopramide**	Prevention and treatment of nausea and vomiting, and management of ileus and delayed gastric emptying.	Dosage 0.25–0.5 mg/kg IV, SC or PO q 8 h or 1–2 mg/kg IV over 24 h as a constant rate infusion (CRI); CRI can be more effective than bolus dosing [92].
**Ondansetron**	For prevention and treatment of vomiting and nausea refractory to other agents such as maropitant, mirtazapine and metoclopramide. Expensive. Injectable or oral formulations available.	Has been used as supportive treatment [6,8,48,64]; use if indicated. Dosage is 0.1–1 mg/kg IV (slowly), SC, or PO q 6–12 h [92].
**Hepatoprotectants such as S-Adenosylmethionine (SAMe)**	Various preparations exist. Uncommonly used but occasional reports of use with antivirals (especially GS-441524) if hepatocellular enzymes (ALT) become elevated [21,37], or when these are normal [19], as protection against hepatic damage; however, ALT normalisation usually occurs rapidly during, or following cessation, of antiviral treatment without the use of hepatoprotectants, so their need is not proven.	Has been used during treatment [19,20,37] without problems. This is an option if clinical concerns exist regarding hepatotoxicity. Some use hepatoprotectants in cases with severe liver enzyme activity elevations but not known if necessary as recovery without is possible.

## 5. Prognosis for Antiviral Treatment Response

Prognostic indicators for response to antivirals are beginning to emerge from studies now that we have effective treatment protocols for FIP.

Negative prognostic indicators, i.e., least likely to survive with treatment, include: high bilirubin [11,23,25,48], elevated lactate dehydrogenase (LDH) activity [26,36,56], sepsis [36], hypoglycaemia [6], jaundice, fever, anaemia and thrombocytopenia [5], higher FCoV nucleocapsid (N) gene RT-PCR loads in abdominal effusion and blood samples [56].

Positive prognostic indicators, i.e., most likely to survive with treatment include: survival for at least 48 h following commencement of antiviral treatment [31] (in one study, 96% of cats that survived 48 h recovered [9]), normalisation of AGP [19], lower bilirubin concentration [23,25] and higher A:G ratio, albumin, α1-, α2- and β-globulin concentrations measured with serum protein electrophoresis (SPE) [56]. A negative blood FCoV N gene RNA RT-qPCR result after two weeks of treatment was also defined as a positive prognostic indicator [56], although this criterion was set as the differentiator for classifying response to treatment, and cats still FCoV N gene RNA PCR-positive after two weeks of treatment had a successful treatment outcome.

## 6. Treatment Monitoring

Monitoring of treatment has mostly been described in cats receiving GS-441524, but the principles apply to other antiviral treatments. Weight gain is a simple measure of treatment efficacy and easy for caregivers to perform at home every one to two weeks using paediatric weighing scales [22,23,25,26,33]. Regular weighing importantly also allows for an increased dose to be calculated to maintain the appropriate dosage despite weight gain in the kitten/cat during recovery [9].

In some cats with FIP and effusions, a pattern of a drop in body weight, a serum globulin concentration upward spike and PCV drop occurs when an effusion is resorbed two weeks into treatment, and this does not indicate treatment failure [9]. Weight loss during the first two weeks of effective treatment has been reported in 21% of cats, interestingly with no difference between cats with or without effusions, although a worsening hyperglobulinaemia in effusive cases was reported [11].

Following resolution of fever and marked improvement of clinical signs, usually within a few days, hyperbilirubinaemia usually resolves quickly, followed by resolution of the effusion (if present, this often resolves within two weeks [56]), then an improvement in haematocrit and increasing A:G ratio. The hyperglobulinaemia can take longer— up to a few weeks—to normalise [8,19]. Cats given molnupiravir treatment can take longer to respond than cats given GS-441524 or remdesivir [47].

If the cat is not hospitalised for supportive care, it is important to obtain a verbal report of progress and ease of medicating the cat from the cat’s caregiver after 48 h of treatment is important as it is essential that antiviral administration is effective. After 2 weeks, any effusions should be reviewed (in-house ultrasound scanning, or abdominal girth measurement as an alternative for abdominal effusion monitoring); if the amount of effusion has not substantially decreased by two weeks, increasing the dosage of the antiviral should be considered (e.g., GS-441524 by 5 mg/kg/day if possible) and/or the diagnosis and differential diagnoses reviewed and/or the formulation of antiviral being used verified. Ideally, haematology and serum biochemistry are repeated after two weeks and then monthly if funds allow, although a minimum is to document normalisation of variables (although mild lymphocytosis and hyperglobulinaemia may still be present after 42 days of treatment) before stopping treatment. Laboratory testing can also be done at a lower cost by measuring only key variables in-house, e.g., total protein/albumin/globulin, bilirubin and/or spinning a microhaematocrit tube for packed cell volume/total proteins/colour of plasma.

AGP concentrations can increase in the first two days of oral GS-441524 treatment before decreasing thereafter [77]. Concentrations of SAA also decrease with effective GS-441524 treatment, even more rapidly than the AGP concentrations (due to the shorter half-life of SAA), mostly reaching normal values by seven days of treatment [77]. Two consecutively normal AGP measurements, at least a week or two apart, might confirm recovery from FIP [19] and be helpful to guide when treatment can be stopped; this can be especially useful now that 42-day treatment courses of GS-441524 are being used. Studies have shown that AGP concentrations decrease continuously after two days of oral GS-441524 treatment, being significantly reduced on day 7 compared to day 1 and usually normalising by days 14–28 of treatment [77].

Clearance of blood FCoV N gene RNA, tested by RT-qPCR, by the second week of treatment was classified as a favourable treatment response in one study; however, cats that were still RT-qPCR-positive in blood by this time also had a successful outcome [56]. FCoV antibody levels are not useful to track response to treatment. Residual abdominal lymphadenomegaly has been reported following effective GS-441524 treatment [5,32,108], as well as a mild lymphocytosis [108], but these do not signify FIP relapse. Residual neurological signs [64,95] are sometimes reported following GS-441524 treatment, but often these signs are static and not associated with FIP relapse. One study [96] described central vestibular signs in three cats that had been successfully treated with GS-441524 over a year before; MRI showed progressive hydrocephalus and no evidence of active FIP was found; these cats were treated with ventriculoperitoneal shunts, and this progressive hydrocephalus might represent post-inflammatory lesions (akin to ‘long COVID-19 syndrome’) beyond viral clearance.

## 7. Preventative Healthcare

In cats in which vaccination and neutering were deemed necessary, both have been performed during, or following, successful treatment of FIP with nucleoside analogues [8,9] without relapse of FIP. However, whether FIP affects vaccine efficacy has not been studied. Individual risk assessments should inform which vaccines are required [109,110,111,112]. Feline-friendly approaches and handling are recommended to minimise stress [89,90], including appropriate analgesia for neutering, gabapentin or pregabalin to reduce stress associated with clinic visits if needed, etc. Some veterinarians advise continuing antiviral treatment for two to four weeks after neutering surgery to ensure antiviral activity is there during a potential period of stress and recovery from surgery [103]. Parasiticide treatments have been administered to cats during and after treatment without problems or concerns regarding efficacy, although published studies are lacking.

## 8. Conclusions

Oral GS-441524 is the most extensively used antiviral for FIP with treatment success rates often exceeding 90% with prompt treatment. Intravenous remdesivir is primarily used in severely affected cats unable to tolerate oral GS-441524 medication, until they are well enough to receive oral medication. In some countries, molnupiravir is readily available and used, although response to treatment may be slower compared to GS-441524. More common adverse effects, including its mutagenic properties, suggest that other nucleoside analogues, such as GS-441524, should be used in preference to molnupiravir, if available. Although 84-day treatment courses have been used historically, shorter protocols of 42 days can be equally effective. Supportive treatments, e.g., analgesia, antiemetics, seizure medications, are important, as well as the rapid treatment of concurrent diseases, e.g., myocarditis, IMHA.

## Data Availability

No new data were created or analysed in this study.
